# Pulse electromagnetic fields enhance extracellular electron transfer in magnetic bioelectrochemical systems

**DOI:** 10.1186/s13068-017-0929-3

**Published:** 2017-10-16

**Authors:** Huihui Zhou, Bingfeng Liu, Qisong Wang, Jianmin Sun, Guojun Xie, Nanqi Ren, Zhiyong Jason Ren, Defeng Xing

**Affiliations:** 1 School of Environment, State Key Laboratory of Urban Water Resource and Environment, Harbin Institute of Technology, 73 Huanghe Road, Nangang District, P.O. Box 2614, Harbin, 150090 Heilongjiang China; 2 School of Electrical Engineering and Automation, Harbin Institute of Technology, Harbin, 150001 China; 3 The Academy of Fundamental and Interdisciplinary Science, Harbin Institute of Technology, Harbin, 150080 China; 4 Department of Civil, Environmental, and Architectural Engineering, University of Colorado Boulder, Boulder, CO 80309 USA

**Keywords:** Magnetic bioelectrochemical system (MBES), Microbial fuel cell, Microbial electrolysis cell, Magnetic field, Pulse electromagnetic field, Magnetic carbon particles, Microbial community

## Abstract

**Background:**

Microbial extracellular electron transfer (EET) is essential in driving the microbial interspecies interaction and redox reactions in bioelectrochemical systems (BESs). Magnetite (Fe_3_O_4_) and magnetic fields (MFs) were recently reported to promote microbial EET, but the mechanisms of MFs stimulation of EET and current generation in BESs are not known. This study investigates the behavior of current generation and EET in a state-of-the-art pulse electromagnetic field (PEMF)-assisted magnetic BES (PEMF-MBES), which was equipped with magnetic carbon particle (Fe_3_O_4_@N-mC)-coated electrodes. Illumina Miseq sequencing of 16S rRNA gene amplicons was also conducted to reveal the changes of microbial communities and interactions on the anode in response to magnetic field.

**Results:**

PEMF had significant influences on current generation. When reactors were operated in microbial fuel cell (MFC) mode with pulse electromagnetic field (PEMF-MMFCs), power densities increased by 25.3–36.0% compared with no PEMF control MFCs (PEMF-OFF-MMFCs). More interestingly, when PEMF was removed, the power density dropped by 25.7%, while when PEMF was reintroduced, the value was restored to the previous level. Illumina sequencing of 16S rRNA gene amplicon and principal component analysis (PCA) based on operational taxonomic units (OTUs) indicate that PEMFs led to the shifts in microbial community and changes in species evenness that decreased biofilm microbial diversity. *Geobacter* spp. were found dominant in all anode biofilms, but the relative abundance in PEMF-MMFCs (86.1–90.0%) was higher than in PEMF-OFF-MMFCs (82.5–82.7%), indicating that the magnetic field enriched *Geobacter* on the anode. The current generation of *Geobacter*-inoculated microbial electrolysis cells (MECs) presented the same change regularity, the accordingly increase or decrease corresponding with switch of PEMF, which confirmed the reversible stimulation of PEMFs on microbial electron transfer.

**Conclusion:**

The pulse electromagnetic field (PEMF) showed significant influence on sta﻿te-of-the-art pulse magnetic bioelectrochemical systems (PEMF-MBES) in terms of current generation and microbial ecology. EET was instantaneously and reversibly enhanced in MBESs inoculated with either mixed-culture or *Geobacter*. PEMF notably decreased bacterial and archaeal diversities of the anode biofilms in MMFCs via changing species evenness rather than species richness, and facilitated specific enrichment of exoelectrogenic bacteria (*Geobacter*) on the anode surface. This study demonstrates a new magnetic approach for understanding and facilitating microbial electrochemical activities.
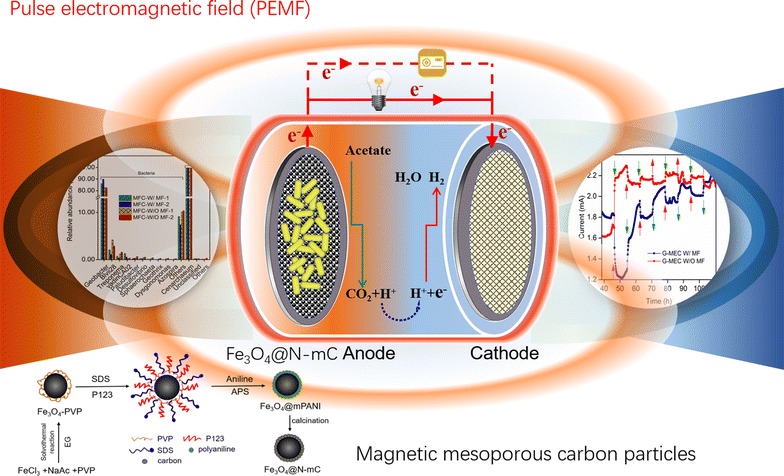

**Electronic supplementary material:**

The online version of this article (doi:10.1186/s13068-017-0929-3) contains supplementary material, which is available to authorized users.

## Background

Bioelectrochemical system (BES) has gained increased attention due to their versatile functions in energy production, environmental remediation, electrosynthesis, and remoting sensing [1–3]. Microbial extracellular electron transfer (EET) is essential in driving the microbial interspecies interaction and redox reactions in both natural and engineered environments. Three types of EET pathways were identified: outer-membrane *c*-type cytochromes, conductive pili, and electron shuttles [4–6], and recent studies found an additional EET pathway mediated by conductive minerals [7]. For instance, magnetite (Fe_3_O_4_) is a conductive mineral that can be used as electron conduits to facilitate EET of Fe(III)-reducing microorganisms. This also compensates for the lack of OmcS to mediate electron transfer between the pili of *Geobacter sulfurreducens* and electron donors or acceptors [8, 9]. Furthermore, electrically conductive magnetite nanoparticles are able to facilitate interspecies electron transfer (IET) between *Geobacter sulfurreducens* and *Thiobacillus denitrificans*, which in turn are able to establish a cooperative metabolism [10]. Nowadays, magnetite has been used for a wide range of applications due to its superparamagnetic properties, low mass transfer resistance, and selective separation of immobilized biomolecules and bacteria by applying magnetic fields (MFs) [11, 12].

Magnetic fields (MFs) provide a cost-effective and convenient approach in changing microbial activity. It has been used in various kinds of MFs-assisted bioreactors [13–15]. Earlier studies demonstrated that MF was able to promote the biocatalytic processes mediated by cytochrome c and to enhance electrical output of the biofuel cells through the magnetohydrodynamics [13, 16]. Recent studies showed that electricity production by MFCs was improved significantly by a static MF [17, 18]. The removal of chemical oxygen demand (COD) in wastewater treatment increases when MFs are applied on MFCs [19]. MFs enhanced bioelectrochemical activities of anodic biofilms, while there was no increase in secretion of redox mediators [20]. Previous studies have shown that MFs enhanced the performance of MFCs. No investigation on how magnetite and MFs influence microbial community structures in MFCs that affect EET. Compared with the permanent magnet, the electromagnetic devices with adjustable intensity and direction of magnetic fields can provide use flexibility for the large-scale BESs. However, the effect of pulse electromagnetic field (PEMF) on BESs has not been investigated.

Electrodes modified with metal oxide, conducting polymer, and nanocomposite have been studied in order to enhance EET and enrichment of exoelectrogenic bacteria [21–26]. However, magnetic material-modified electrodes have not been applied in BESs. Some properties of magnetite (Fe_3_O_4_) are sensitive to oxidation, and agglomeration and low conductivity makes it less than ideal. To circumvent this, Fe_3_O_4_ has been used as hybrid material with various other materials that enhances the properties of the magnetic particles. Polyaniline (PANI) is a carbon–nitrogen precursor that is popular within carbon material preparation because they have an aromatic structure that is conductive to the formation of graphitization structure after carbonization, and it was shown to improve the performance of MFCs as the conductive polymer [27–29] by serving as a conductive and protective shell for magnetite in Fe_3_O_4_/polyaniline hybrid [12, 30, 31].

In this study, we hypothesized that the magnetic carbon particle-modified electrodes in BESs may facilitate EET and current production under pulsed magnetic fields. To test this hypothesis, we constructed a new magnetic bioelectrochemical system (MBES) with magnetic carbon particle-modified electrodes, and we developed a pulse electromagnetic field (PEMF) to hopefully enhance microbial extracellular electron transfer. Furthermore, we used molecular biology tools to understand the changes of microbial communities and interactions on the anode in response to PEMF operation in the PEMF-MBES reactors.

## Methods

### Synthesis of Fe_3_O_4_@N-mC composite

The fabrication of Fe_3_O_4_@N-mC is previously described (Additional file 1: Fig. S1) [31]. Fe_3_O_4_@N-mC was the product of the carbonization of magnetic mesoporous polyaniline composite (Fe_3_O_4_@mPANI), and the Fe_3_O_4_@mPANI was obtained from aniline polymerization on the surface of the PVP-modified Fe_3_O_4_ particles (Fe_3_O_4_-PVP). Fe_3_O_4_-PVP was synthesized using the solvothermal method [32]. Ferric chloride hexahydrate, sodium acetate, and PVP were dissolved in ethylene glycol, and then crystallized at 200 °C for 8 h. Synthesized Fe_3_O_4_-PVP (black magnetic particles) was separated and recovered using a strong magnet. Fe_3_O_4_-PVP was added to the mixture of tergitol(tm)xh(nonionic)(P123) and sodium dodecyl sulfate (SDS) and dissolved in diluted hydrochloric acid (1 mol/L). After 30 min, it was ultrasonically dispersed, and the mixture was placed at 4 °C. The mixture was stirred, and aniline (0.1 mol/L) and ammonium persulfate (0.4 mol/L) were slowly added into the mixture. After that, the mixture was stirred for an additional 6 h to obtain Fe_3_O_4_@mPANI. Finally, the Fe_3_O_4_@mPANI was carbonized with nitrogen at a heating rate of 3 °C min^−1^ to 700 °C and kept at 700 °C for 6 h in a tube furnace to synthesize Fe_3_O_4_@N-mC.

### Configuration and operation of magnetic BESs

Single-chamber microbial fuel cells (MFCs) (cylindrical chamber, 28 mL) were constructed, as previously described [33]. The anode was stainless steel mesh (SSM, type SUS304) coated with Fe_3_O_4_@N-mC (5 mg/cm^2^). The cathode rolled the activated carbon-PTFE air cathode [34]. The effective area of each cathode and anode was 7 cm^2^, respectively, which were connected by titanium wires through an external resistance of 1000 Ω. The wires on the electromagnetic launcher were intertwined on the external walls of the anode chamber in order to obtain a pulse electromagnetic field (symmetric periodic square wave) with the frequency of 100 Hz. A magnetic intensity of 5 uT was applied on the reactors as they were 5 cm away from electric magnetic field analyzer (EHP-200A, Narda, Inc., Italy). Activated sludge from a wastewater treatment plant (Harbin, China) was used to inoculate the magnetic MFCs (MMFCs). MMFCs were operated in both the presence (PEMF-MMFCs) and absence of PEMFs (PEMF-OFF-MMFCs). The cultured solution contained (per liter) 2 g sodium acetate, 50 mM phosphate buffer solution (PBS) (11.55 g/L Na_2_HPO_4_·12H_2_O, 2.77 g/L NaH_2_PO_4_·2H_2_O, 0.31 g/L NH_4_Cl and 0.13 g/L KCl), 10 mL mineral solution, and 10 mL vitamin solution [35]. For each test, there was a duplicate MMFC in operation in the fed-batch mode at a constant temperature (25 ± 2 °C).

For the pure culture test, single-chamber microbial electrolysis cells (MECs) were constructed using a 250-mL anaerobic bottle with a liquid volume of 100 mL. The anode was carbon paper and the cathode rolled activated carbon-PTFE. The effective area of each cathode and anode was 5.4 cm^2^ (1.8 cm × 3 cm), respectively. A fixed voltage of 0.6 V was applied to MECs by a programmable power source (3645A, Array, Inc.). All reactors were inoculated with *Geobacter sulfurreducen*s PCA (ATCC 51573) purchased from American Type Culture Collection (ATCC). MECs were filled with the medium containing (per liter, pH 6.8): 0.82 g sodium acetate, 0.1 g KCl, 1.5 g NH_4_Cl, 0.6 g NaH_2_PO_4_, 2 g NaHCO_3_, 10 mL mineral solution, and 10 mL vitamin solution. Prior to use, the medium was flushed with N_2_-CO_2_ (80:20). All MECs were operated at a constant temperature (35 ± 2 °C). The pulse electromagnetic fields were applied on MECs by a sequential “ON” and “OFF” process to analyze the instantaneous effect on the current generation by MECs. The current in the circuit was conducted by measuring the voltage across a high-precision resistor (10 Ω).

### Analytical and electrochemical techniques

MMFC voltage over the external resistance was automatically recorded every 5 min by a multi-channel data acquisition system (Model 2700 with 7702 module, Keithley Instruments Inc., USA). Both linear sweep voltammetry (LSV) and electrochemical impedance spectroscopy (EIS) were measured on the two-electrode mode. This analyzed the entire cell with the anode as the working electrode and the cathode as the reference electrode by using Autolab Potentiostat/Galvanostat (Autolab PGSTAT 128 N, MetrohmAutolab Inc., Netherlands). Polarization and power density curves were plotted based on linear sweep voltammetry (LSV), which was conducted from open-circuit voltage (OCV) to 0 V with a scan rate of 0.1 mV/s. Electrochemical impedance spectroscopy (EIS) was conducted with a frequency range of 100 kHz–10 mHz at the OCV of MFCs. The Nyquist plots were analyzed using NOVA software. The impedance spectra were analyzed by fitting to the equivalent circuit (EC): *R* (Q [RW]). The EC contained *R*_s_, *R*_p_, *W*, and CPE four parts. *R*_s_ represents ohmic resistance; *R*_p_ represents activation resistance; constant phase element (CPE) represents the electrical double-layer capacitor; and Warburg (W) represents diffusion resistance [36].

X-ray diffraction (XRD) characterization was obtained on a Bruker AXS D8-Advanced diffractometer with CuKα radiation (*λ* = 1.5418 Å). Raman experiments were performed on a LabRAM XploRA Raman microscope using a 532 nm excitation line from an argon-ion laser with a power of 0.15 mW. The magnetization curve was carried out on Quantum Design MPMS-7 SQUID magnetometer at 300 K under varying magnetic fields. The samples were analyzed by Fourier transform infrared spectroscopy (FTIR) on Perkin Elmer 100 spectrometer from 400 to 4000 cm^−1^ and X-ray photoelectron spectroscopy (XPS; PH1-5700 ESCA system, US) using a hemispherical analyzer and an aluminum anode (monochromatic Al Ka, 1486.6 eV) as the source (at 12–14 kV and 10–20 mA). The microstructures of samples were observed by scanning electron microscope (SEM; Helios Nanolab600i, FEI, USA) and transmission electron microscope (TEM; JEM-2100 electron microscope, JEOL, Japan) [37].

### Illumina sequencing of 16S rRNA gene amplicons

After 2 months of operation, the anodes of MMFCs were cut and fragmented using sterile scissors [38]. Genomic DNA of the anode biofilms were extracted using the PowerSoil DNA Isolation Kit (Mo Bio Laboratories, Inc., Carlsbad, CA) according to the manufacturer’s instructions. DNA from two pieces of an electrode were isolated and mixed equally for PCR amplification after DNA concentration was determined using Qubit fluorometer (Thermo Fisher Scientific). V4–V5 regions of bacterial and archaeal 16S rRNA genes were amplified using universal primers: 515F (5′-GTGCCAGCMGCCGCGGTAA-3′) and 907R (5′-CCGTCAATTCCTTTGAGTTT-3′); U519F (5′-CAGYMGCCRCGGKAAHACC-3′) and 806R (5′-GGACTACNSGGGTMTCTAAT-3′). PCR products were examined using agarose gel electrophoresis and then purified using a PCR purification kit (Qiagen, Germany). High-throughput sequencing of 16S rRNA gene amplicons was carried out on the Illumina HiSeq 2500 platform.

The sequencing reads were initially assembled into raw tags. Effective reads were obtained after the raw reads were trimmed, filtered, and removed chimeric sequences using QIIME software (http://qiime.org). Operational taxonomic units (OTUs) were determined at a 97% sequence similarity cutoff using UPARSE software [39]. The representative OTUs were selected according to the highest frequency and assigned to a taxonomic identification by using the RDP Classifier at a 0.8 confidence threshold [40]. Species diversities (Observed-species, Chao1, Shannon, Simpson, and ACE) were calculated by QIIME software.

## Results

### Synthesis and characterization of Fe_3_O_4_@N-mC modified electrodes

**Fig. 1:**
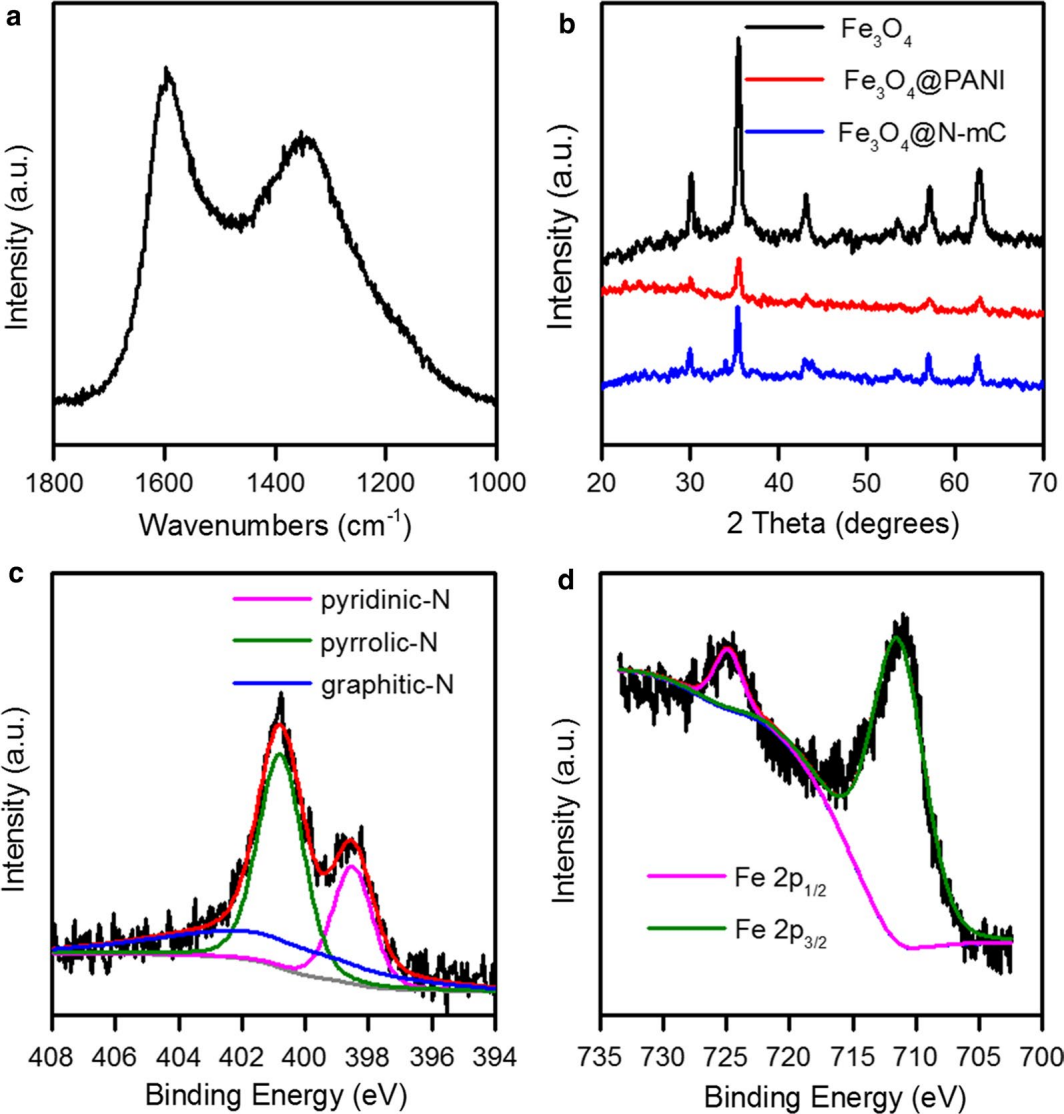
Characterizations of synthesized Fe_3_O_4_@N-mC using Raman spectra (**a**), XRD (**b**), and high-resolution XPS [N 1s (**c**) and Fe 2p (**d**)]

A well-defined core–shell structure of Fe_3_O_4_@N-mC composite was synthesized. As shown in TEM micrographs, a dark inner core (Fe_3_O_4_) was coated with a lighter carbon skin, and SEM images showed that the shell contained by many tiny nanoparticles around 30 nm in length (Additional file 1: Fig. S2). The Raman spectrum of Fe_3_O_4_@N-mC showed two peaks at around 1330 and 1600 cm^−1^ that could be labeled as the D-band and G-band of disordered and graphitized carbons (Fig. 1a) [41], demonstrating that the carbon structure existed on the surface of Fe_3_O_4_.

The XPS survey spectrum of Fe_3_O_4_@N-mC confirmed the existences of C, O, N, and Fe elements; the element contents were also measured (Additional file 1: Fig. S3). The high-resolution N 1s scan revealed three typical N species, namely pyridinic N (398.5 eV), pyrrolic N (400.4), and graphitic N (401.3) (Fig. 1c) [42]. The FTIR characteristic peaked at 1590 cm^−1^ thus belonging to the –NH_2_ deformation vibration. This also indicated the successful N-functionalization on the Fe_3_O_4_ surface [43]. Peaks at 1605, 1494, 1296, 1235, and 1148, 830 cm^−1^ were in good agreement with PANI [44], which disappeared in Fe_3_O_4_@N-mC due to the carbonization process. Additionally, two peaks at 470 and 585 cm^−1^ were categorized as the Fe–O stretching vibrations of Fe_3_O_4_ (Additional file 1: Fig. S4) [45]. The XRD patterns of Fe_3_O_4_, Fe_3_O_4_@PANI, and Fe_3_O_4_@N-mC showed five significant diffraction peaks that were in good agreement with cubic lattice of Fe_3_O_4_ (JCPDS No. 19-0629) (Fig. 1b). When Fe_3_O_4_ was coated with a layer of PANI, the intensity of its diffraction peaks decreased, and the intensity of the peaks increased after the carbonization. The standard S-shaped hysteresis curve of the Fe_3_O_4_@N-mC depict its magnetic behavior (Additional file 1: Fig. S5). Fe 2p high-resolution spectrum indicated that Fe species were in the form of Fe 2p_1/2_ (725 eV) and Fe 2p_3/2_ (711.3 eV) (Fig. 1d) [46]. These results are proof that the Fe_3_O_4_@N-mC composite was successfully synthesized.

### PEMF enhanced current generation by MMFCs

**Fig. 2:**
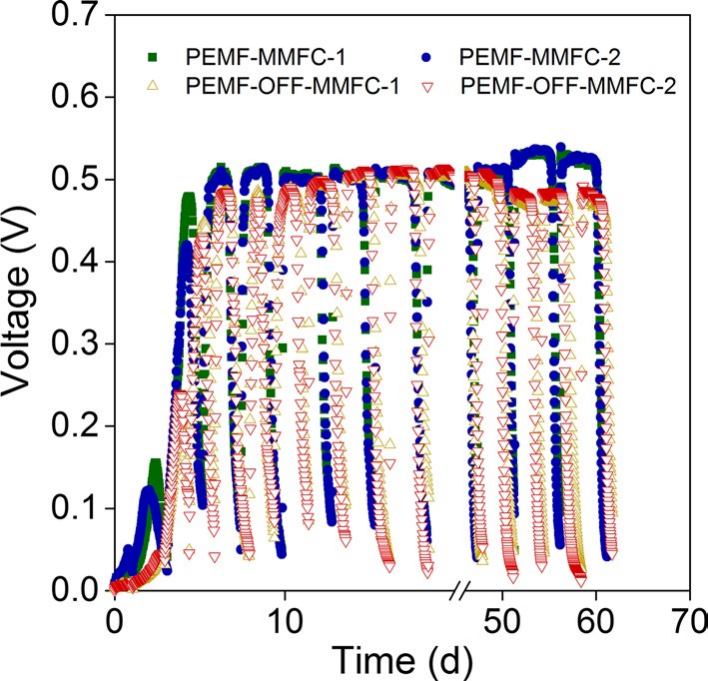
Voltage production of MMFCs in the presence (PEMF-MMFCs) and absence of PEMF (PEMF-OFF-MMFCs) at external resistance of 1000 Ω. Numbers represent duplicate MMFC reactors

The performance of magnetic MFCs (MMFCs) equipped with Fe_3_O_4_@N-mC-coated anodes was characterized in the presence or absence of PEMF. MMFCs exposed to PEMF (PEMF-MMFCs) started up faster as its voltage increased rapidly at 60 h compared with the MFCs without PEMF (PEMF-OFF-MMFCs) (95 h), indicating that MF accelerated the start-up of MMFCs (Fig. 2). The peak voltage of PEMF-MMFC (508 mV,1000 Ω of external resistance) was slightly higher than that of PEMF-OFF-MMFC (485 mV) during the initial start-up stage. MFCs had a similar peak voltage (503 mV) during the stable operation stage (12–50 days). The peak voltage of PEMF-MMFC increased to 529 mV after the long-term operation, versus 483 mV of PEMF-OFF-MMFC. PEMF-MMFCs demonstrated a longer discharge period of batch cycle (~ 118 h) than PEMF-OFF-MMFCs (~ 78 h) and higher peak voltage. This suggests that exoelectrogenic biofilms in PEMF-MMFCs had a higher capability of extracellular electron transfer than those in PEMF-OFF-MMFCs.

**Fig. 3:**
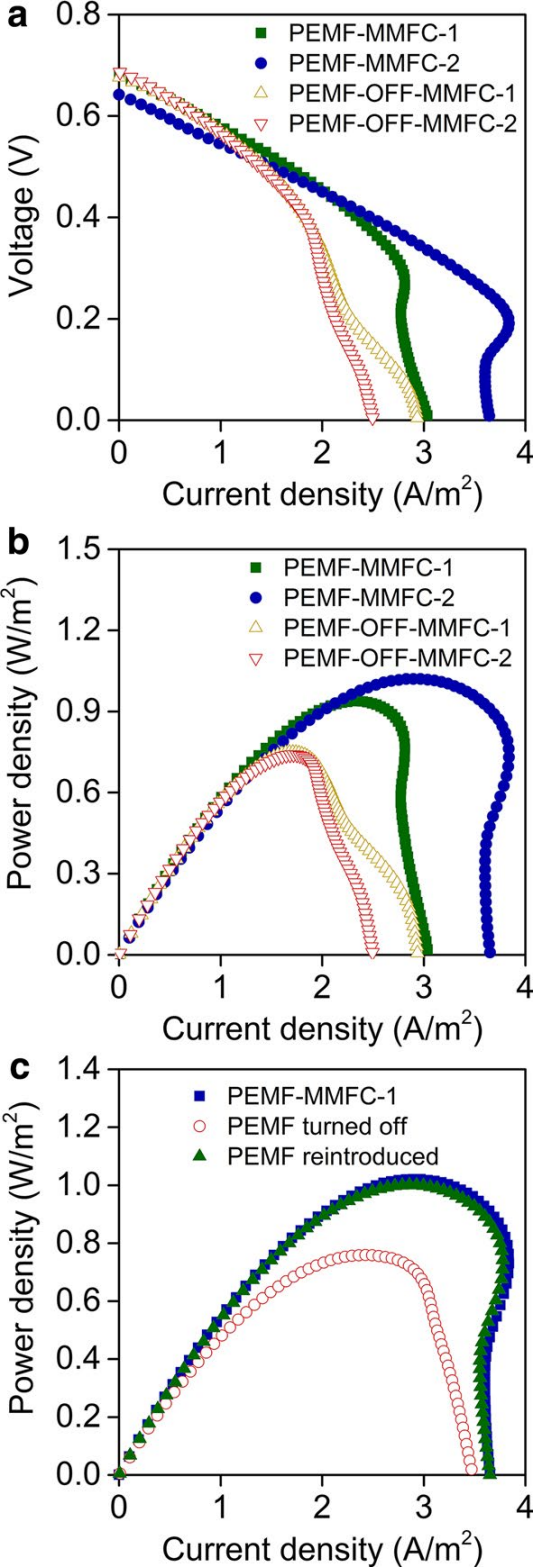
Polarization (**a**) and power density (**b**, **c**) curves of MMFCs under the on and off cycle of PEMF

In the medium and high current density regions of polarization curves, PEMF-MMFC showed higher potential than PEMF-OFF-MMFC when current densities were the same, suggesting a lower activation resistance and a lower diffusion resistance of PEMF-MMFC (Fig. 3a). PEMF-MMFC obtained the highest maximum power densities of 0.94–1.02 W/m^2^, which is 25.3–36.0% higher than the 0.74–0.75 W/m^2^ obtained by PEMF-OFF-MMFC (Fig. 3b). The maximum power density of PEMF-MMFC decreased 25.7% when PEMF was turned off, which was then restored perfectly when PEMF was reintroduced (Fig. 3c).

**Fig. 4:**
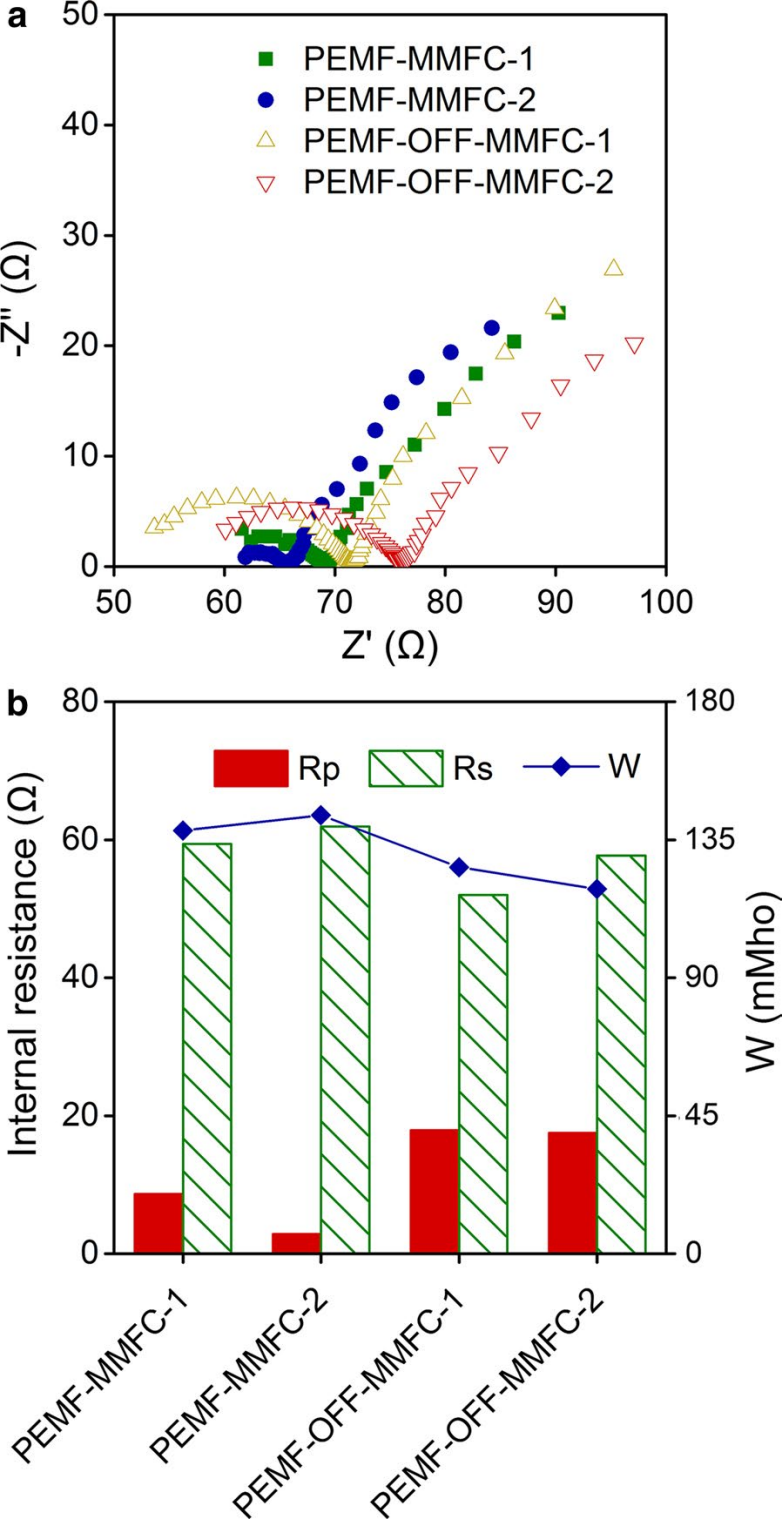
Nyquist plots (**a**) and internal resistances of PEMF-MMFCs and PEMF-OFF-MMFCs analyzed by fitting to the equivalent circuit: R (Q [RW]) (**b**)

On the basis of the Ohm’s law, the internal resistances of MMFCs were calculated in Additional file 1: Table S1. PEMF-MMFCs showed lower internal resistance of 61.4–69.6 Ω when compared to PEMF-OFF-MMFCs (87.2–88.0 Ω), indicating the addition of PEMF resulted in the decrease of the internal resistance of MMFCs. According to electrochemical impedance spectroscopy (EIS) analysis, PEMF-MMFCs had similar ohmic resistance as PEMF-OFF-MMFCs, but its activation resistance ranged from 2.83 to 8.65 Ω, much lower than the range of 17.5–17.9 Ω measured for PEMF-OFF-MMFCs (Fig. 4). PEMF-MMFC had lower diffusion resistance since its conductivity of 138–143 mho was higher than 119–126 mho of PEMF-OFF-MMFC. Thus, PEMF decreases the internal resistance of MMFCs by reducing its activation resistance and diffusion resistance.

### PEMF and magnetic anode influenced bacterial and archaeal community structures

**Fig. 5:**
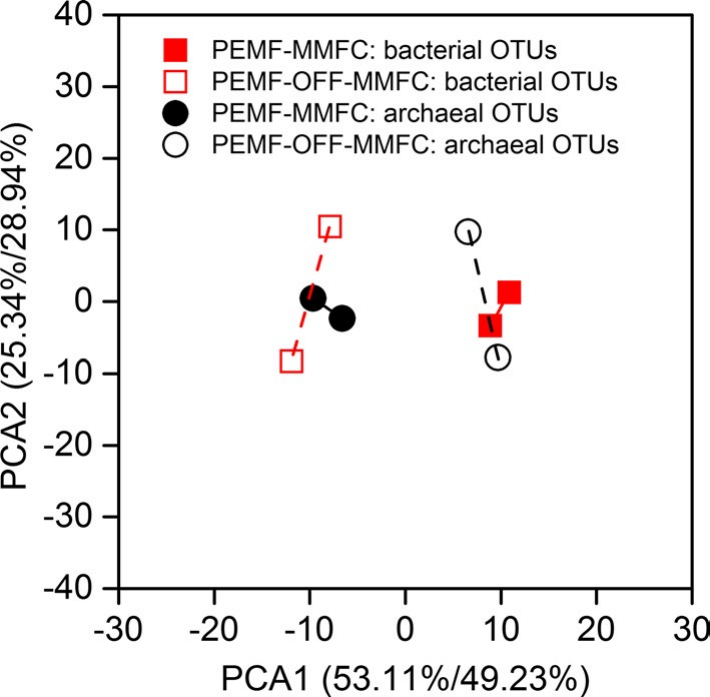
Principle component analysis (PCA) based on bacterial and archaeal operational taxonomic units of the anode biofilms of PEMF-MMFCs and PEMF-OFF-MMFCs

Reads were obtained from each sample after filtering and removing chimera (Additional file 1: Table S2), ranging from 37,730 to 59,939 with a length of 373. The Good’s coverage estimator of 0.999–1 indicated that almost the entire microbial community for each sample was tracked using Illumina sequencing of 16S rRNA gene amplicons. Total operational taxonomic units (OTUs) of 146 and 141 in bacteria, along with 95 and 114 in archaea were detected for the anode biofilms of PEMF-MMFCs at a threshold of 97%, with OTUs of 149 and 164 in bacteria, and 115 and 128 in archaea for the anode biofilms of PEMF-OFF-MMFCs. The observed OTUs and expected richness (Chao1 and ACE estimators) were similar between both the PEMF-OFF-MMFC-1 and the PEMF-MMFC-1; however, the Shannon’s and the Simpson’s diversity indices of PEMF-MMFCs were lower than that of PEMF-OFF-MMFCs. This suggests that the application of PEMF on MMFCs resulted in the decrease in species evenness, rather than species richness. Principal component analysis (PCA) showed that there were distinct separations of bacterial and archaeal communities between PEMF-MMFCs and PEMF-OFF-MMFCs, indicating that PEMF led to significant shifts in the microbial community structures (Fig. 5).

### PEMF and magnetic anode facilitated the enrichment of exoelectrogenic bacteria

**Fig. 6:**
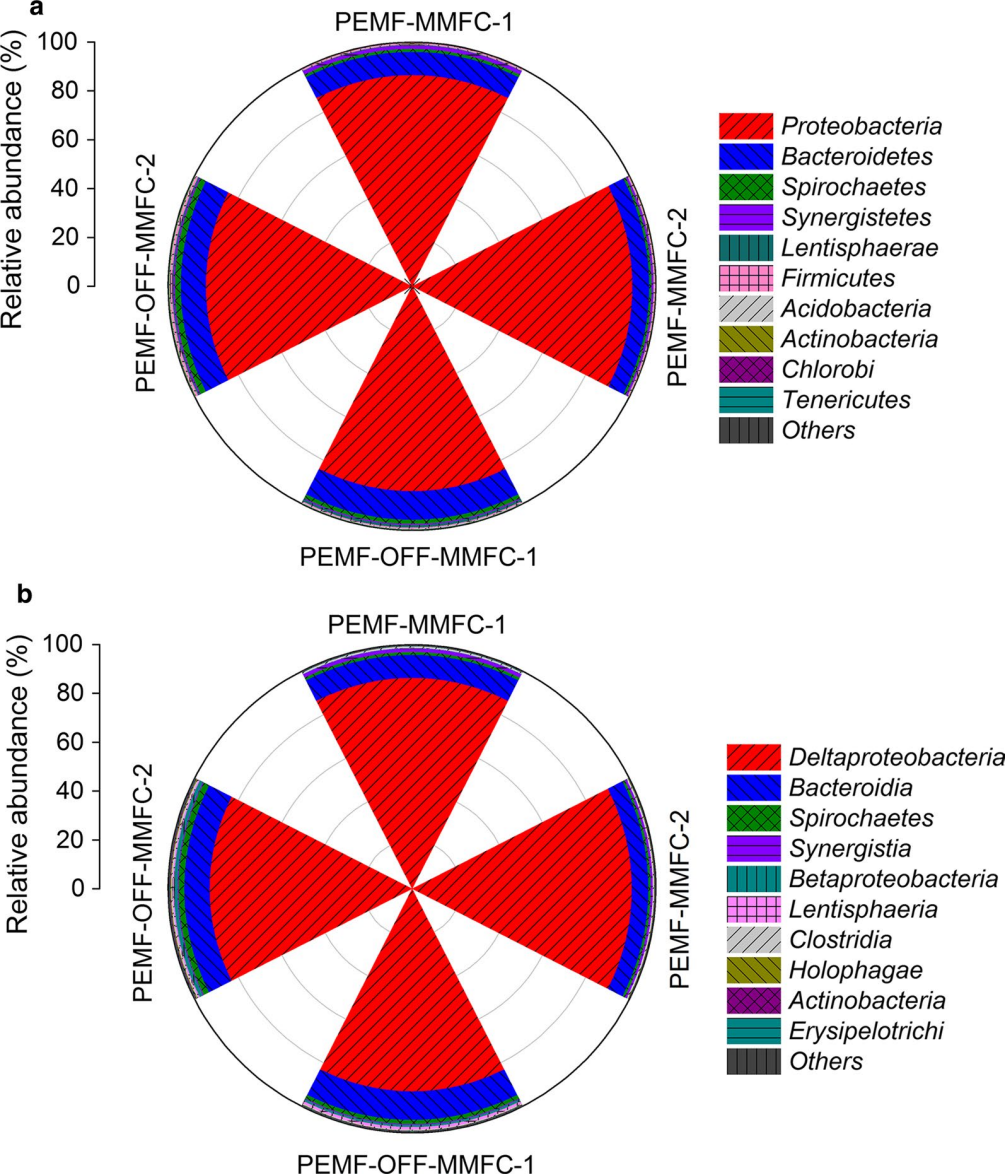
Taxonomic classification of bacterial 16S rRNA sequences from bacterial communities of PEMF-MMFCs and PEMF-OFF-MMFCs at the phylum level (**a**) and class level (**b**). The relative abundances of the top 10 phyla and classes are shown

**Fig. 7:**
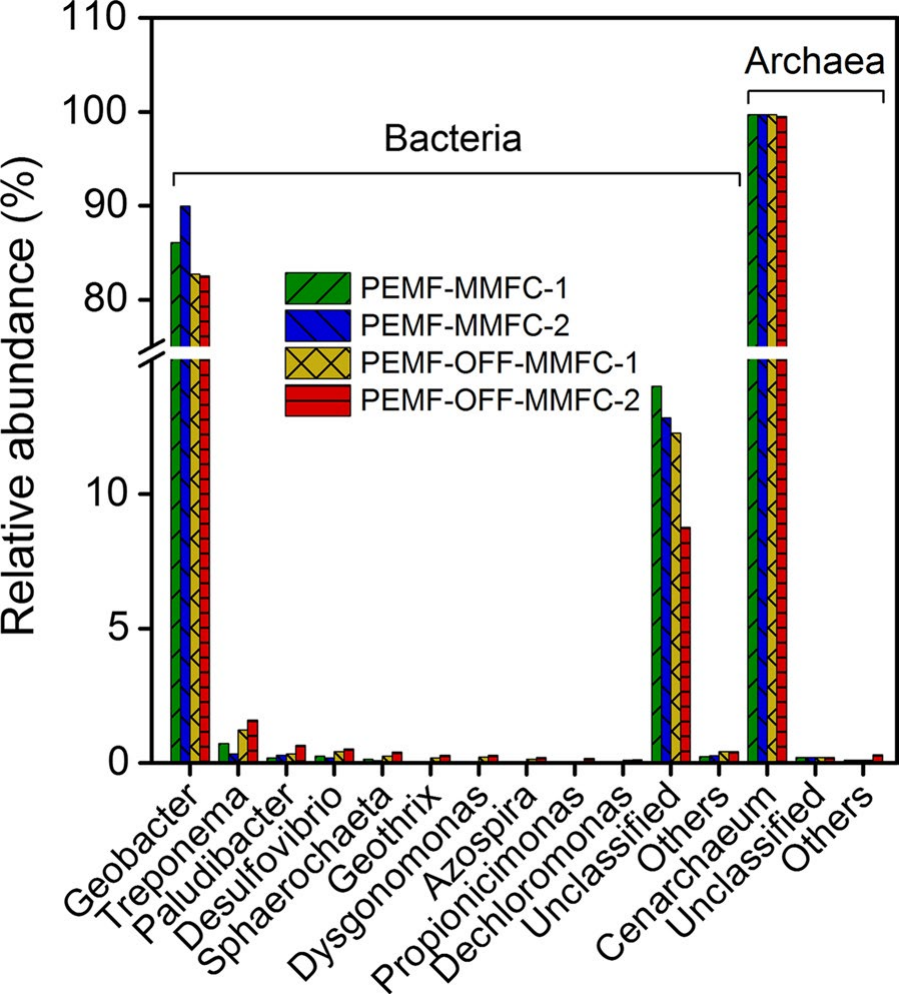
The relative abundance of predominant bacterial and archaeal populations in the anode biofilms of PEMF-MMFCs and PEMF-OFF-MMFCs. The relative abundances of the top 10 genera are shown

The predominant phyla present were *Proteobacteria* and *Bacteroidetes* in all obtained samples (Fig. 6a). The relative amount of *Proteobacteria* in PEMF-MMFCs (86.7 and 90.5%) was slightly higher than that in PEMF-OFF-MMFCs (84.2 and 84.7%). However, the relative abundance of *Bacteroidetes* in PEMF-MMFCs (6.9 and 9.5%) was lower than that in PEMF-OFF-MMFCs (10.3 and 11.7%). At class level, the relative abundance of *Deltaproteobacteria* (86.4 and 90.2%) in PEMF-MMFCs was higher than that in PEMF-OFF-MMFCs (83.1 and 83.2%) (Fig. 6b). The majority of predominant populations were affiliated with *Geobacter* in MMFCs (Fig. 7). The relative abundance of *Geobacter* in PEMF-MMFCs (86.1 and 90.0%) was higher than that in PEMF-OFF-MMFCs (82.5 and 82.7%), implying that PEMF enabled enrichment of exoelectrogens on the anode. Archaeal communities in MMFCs were dominated by *Cenarchaeum symbiosum,* accounting for 99.5–99.8% of the archaea, indicating that PEMF did not predominantly shift archaeal populations, although it did shift the species diversity of archaeal communities.

### PEMF reversibly stimulated current generation by *Geobacter*

**Fig. 8:**
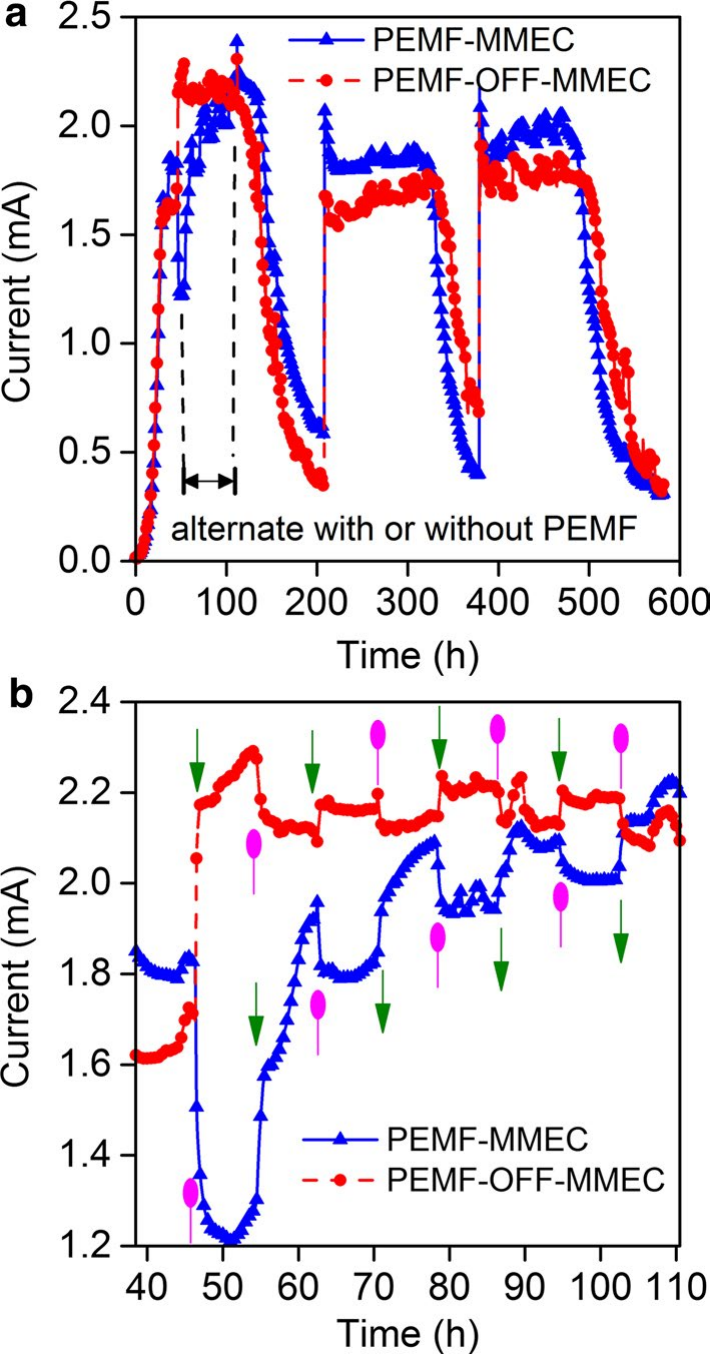
Current generation by *Geobacter sulfurreducens* PCA in MECs under the on and off cycle of PEMF. **b**  presents expanding curves of current generation in MECs during switching of PEMF in an operation cycle (between dish lines in **a**). The green triangle and pink ellipse arrows represent turn on and off of PEMF (**b**)

Since the predominant population in all anode biofilms was affiliated with *Geobacter sulfurreducens* (similarity of 99–100%), the effects of PEMF on current generation by *G. sulfurreducens* in microbial electrolysis cells (MECs) were evaluated. Current generation by *Geobacter* in PEMF-MMEC was higher than that in PEMF-OFF-MEC (Fig. 8a). In order to evaluate instantaneous response of *Geobacter* to PEMF during steady operation stage, PEMF was periodically turned off (PEMF-OFF-MEC) and on (PEMF-MEC) at 8-h interval. Results show that current generation by *Geobacter* corresponded very well with the PEMF cycle, during which current increased with the application of PEMF and decreased with the absence of PEMF. Interestingly, PEMF effectively stimulated current generation by *Geobacter* in MECs, no matter the initial MECs were operated in the presence or absence of PEMF (Fig. 8b).

## Discussion

### Pulse electromagnetic field stimulated magnetic BES (PEMF-MBES)

This study reports for the first time that pulse electromagnetic field (PEMF) stimulated current generation in magnetic BES (MBES) with Fe_3_O_4_@N-mC-coated electrode (PEMF-MBES). Magnetic MFCs (MMFCs) that were exposed to PEMF presented fast biofilm acclimation, and the maximum power density increased by 25.3–36.0% compared with MMFCs without PEMF (Fig. 3b). Such stimulation was further confirmed by reversible effects of PEMF, where power output was decreased by 25.7% when PEMF was off and resumed to previous level when it was backed on. This phenomenon where PEMF prolonged the discharge period of batch cycle and enhanced the voltage output was consistent with the previous study on the effect of permanent magnetic field on MFCs [20]. Polarization curves showed that PEMF was unable to enhance the potential of open-circuit MFCs (Fig. 3a), implying that PEMF impacted the activity of the anode biofilms. The electrochemical impedance spectroscopy (EIS) analyses showed that when PEMF was present there was a decrease in the activation losses and mass transfer losses (Fig. 3b), which facilitated system mass transfer and consistent with previous studies [18]. Reversible effects of PEMF on current generation by BES provided an instant and controllable method to stimulate EET, which is important for revealing the mechanism of EET.

Recent studies showed that centimeter-long electron transport in marine sediments via conductive minerals is possible [47]. This suggests that the planktonic microbes in the solution of MECs can be trapped and immobilized by magnetic carbon nanoparticles (Fe_3_O_4_@N-mC) and deposited around the anode by controlling magnetic field based on the enhancement capacity requirements of EET. Magnetic Fe_3_O_4_@N-mC may also be used as the cathode catalyst. Thus, to improve PEMF-MBES, the effects of magnetic intensity and pulse repetition frequency of PEMF and magnetic carbon nanoparticles on extracellular electron transfer of exoelectrogens need to be investigated in future studies.

### PEMF led to shifts in bacterial community diversity in MMFCs

Although several studies have reported the effects of permanent magnetic field on electricity generation in MFCs, this study investigates the response of microbial community to magnetic fields and elucidates the ecological response that resulted in the improved performance. The diversity indices show an obvious decrease in bacterial and archaeal diversity of the anode biofilms when PEMF was present, although it seems that it decreased species evenness rather than species richness.

The relative abundance of *Geobacter* in MMFCs with Fe_3_O_4_@N-mC went up to 82.5–90.0% (Fig. 7), implying that Fe_3_O_4_@N-mC facilitated specific enrichment of *Geobacter* on the anode. Previous studies showed that magnetite supplied to rice paddy field soil promoted a specific enrichment of *Geobacter* species and as well as current generation [8]. A recent study also reported that magnetic particle facilitated enrichment of exoelectrogenic bacteria [48]. The relative abundance of *Geobacter* in PEMF-MMFCs was 6.6% higher than that in PEMF-OFF-MMFCs, suggesting that PEMF enhanced the enrichment of *Geobacter* and therefore produced higher power. Therefore, PEMF and Fe_3_O_4_@N-mC coatings are able to shape microbial community structures of the anode biofilms by enriching exoelectrogenic bacteria.

*Cenarchaeum symbiosum,* an uncultivated archaeon, was abundant in the anode biofilms (99.5–99.8% of relative abundance), which is the sole archaeal symbiont of the marine sponge *Axinella Mexicana* [49]. Investigating its presumable capacity of extracellular electron transfer of *Cenarchaeum symbiosum* and its role in the anode biofilms would be significant in understanding exoelectrogenic microorganisms.

### The mechanisms of PEMF stimulation for EET in MBES

This is the first evidence that *Geobacter sulfurreducens* PCA is able to respond immediately to PEMF (Fig. 8). Another well-known exoelectrogen, *Shewanella oneidensis* MR-1, showed a similar increase in current generation in MFCs exposed to permanent MF but was unable to significantly enhance mediator secretion [17]. Both pure- and mixed-culture tests have indicated that the effect of PEMF on current generation is instantaneous and reversible; we speculate that PEMF directly enhance capacity of extracellular electron transfer due to the changes in the electroactive activities of cytochrome c and redox enzyme through the magnetohydrodynamic effect, but further evidence is needed in follow-up studies. Electromagnetohydrodynamic effect might reduce the activation resistance and diffusion resistance by affecting the electron motion according to the Lorentz force law. PEMF may also indirectly promote electron transfer by assembling dispersed conductive minerals around exoelectrogenic bacteria. Recent studies reveal that the manganic Fe_3_O_4_ effectively promotes extracellular electron transfer in a manner similar to c-type cytochromes [9, 10]. To gain insight into the mechanisms of the PEMF stimulation for extracellular electron transfer, the relationships among EET, magnetohydrodynamic effect, pulse frequency and expression and activity of cytochrome and redox enzyme of the exoelectrogenic biofilms need further investigation.

## Conclusions

The pulse electromagnetic field (PEMF) significantly influenced current generation and community deversity of magnetic bioelectrochemical system (PEMF-MBES). The PEMF stimulated current generation when it was applied to MMFCs with magnetic mesoporous carbon particle-coated anode. MBESs inoculated with either mixed-culture or *Geobacter* showed similar responses under PEMF, in which extracellular electron transfer was instantaneously and reversibly enhanced. Illumina sequencing of 16S rRNA gene amplicons demonstrated that PEMF notably decreased bacterial and archaeal diversities of the anode biofilms in MMFCs via changing species evenness rather than species richness. The relative abundance of *Geobacter* was higher in the presence of PEMF, indicating that PEMF-MMFC facilitated specific enrichment of *Geobacter* on the anode surface.

## Additional file

**Additional file 1: Table S1.** Comparison of performance of PEMF-MMFCs and PEMF-OFF-MMFCs. **Table S2.** Similarity-based OTUs and species richness and diversity indices at a threshold of 97%. **Figure S1.** Schematic illustration of the synthesis of Fe_3_O_4_@N-mC composite. **Figure S2.** TEM (a) and SEM (b) images of synthesized Fe_3_O_4_@N-mC composite. **Figure S3.** Survey XPS spectra of synthesized Fe_3_O_4_@N-mC composite. The insert represents element contents. **Figure S4.** FTIR spectra of Fe_3_O_4_, Fe_3_O_4_@mPANI and Fe_3_O_4_@N-mC composite. **Figure S5.** Magnetic hysteresis loops of Fe_3_O_4_ and of synthesized Fe_3_O_4_@N-mC.

## Abbreviations

MFs: magnetic fields
EET: extracellular electron transfer
BESs: bioelectrochemical systems
PEMF: pulse electromagnetic field
PEMF-MBES: pulse electromagnetic field-assisted magnetic BES
Fe_3_O_4_-PVP: PVP-modified Fe_3_O_4_ particles
Fe_3_O_4_@mPANI: PANI-modified Fe_3_O_4_ particles
Fe_3_O_4_@N-mC: magnetic nitrogen-doped carbon particle
MFC: microbial fuel cell
PEMF-MMFCs: pulse electromagnetic field-assisted magnetic microbial fuel cell
PEMF-OFF-MMFCs: no pulse elctromagnetic field control magnetic microbial fuel cell
PCA: principal component analysis
OTUs: operational taxonomic units
MECs: microbial electrolysis cells
IET: interspecies electron transfer
COD: chemical oxygen demand
PANI: polyaniline
LSV: linear sweep voltammetry
EIS: electrochemical impedance spectroscopy
OCV: open-circuit voltage
XRD: X-ray diffraction
FTIR: fourier transform infrared spectroscopy
XPS: X-ray photoelectron spectroscopy
SEM: scanning electron microscope
TEM: transmission electron microscope
P123: tergitol(tm)xh(nonionic)
SDS: sodium dodecyl sulfate
