# Supplementary material for: Pulse electromagnetic fields enhance extracellular electron transfer in magnetic bioelectrochemical systems
Source: Biotechnol Biofuels. 2017 Oct 16;10:238. doi: 10.1186/s13068-017-0929-3 (PMC5644122; doi:10.1186/s13068-017-0929-3)
Supplement: Supplementary file 1 — Additional file 1: Table S1. Comparison of performance of PEMF-MMFCs and PEMF-OFF-MMFCs. Table S2. Similarity-based OTUs and species richness and diversity indices at a threshold of 97%. Figure S1. Schematic illustration of the synthesis of Fe3O4@N-mC composite. Figure S2. TEM (a) and SEM (b) images of synthesized Fe3O4@N-mC composite. Figure S3. Survey XPS spectra of synthesized Fe3O4@N-mC composite. The insert represents element contents. Figure S4. FTIR spectra of Fe3O4, Fe3O4@mPANI and Fe3O4@N-mC composite. Figure S5. Magnetic hysteresis loops of Fe3O4 and of synthesized Fe3O4@N-mC. [file 13068_2017_929_MOESM1_ESM.pdf]

Page: 4; Table: 2; Figure: 5

Supplementary Information

The following information is provided to the article in

Biotechnology for Biofuels

on

"Pulse electromagnetic fields enhance extracellular electron transfer in magnetic bioelectrochemical systems"

Huihui Zhou [1], Bingfeng Liu[1], Qisong Wang[2], Jianmin Sun[1, 3], Guojun Xie[1], Nanqi Ren[1], Zhiyong Jason Ren[4], Defeng Xing[1*]

[1]School of Environment, State Key Laboratory of Urban Water Resource and Environment, Harbin Institute of Technology, Harbin 150090, China

[2]School of Electrical Engineering and Automation, Harbin Institute of Technology, Harbin 150001, China

[3]The Academy of Fundamental and Interdisciplinary Science, Harbin Institute of Technology, Harbin 150080, China

[4]Department of Civil, Environmental, and Architectural Engineering, University of Colorado Boulder, Boulder, Colorado 80309, United States

[*] **Corresponding author**. School of Municipal and Environmental Engineering, Harbin Institute of Technology, P.O. Box 2650, 73 Huanghe Road, Nangang District, Harbin, Heilongjiang Province 150090, China

E-mail address: dxing@hit.edu.cn

Tel& Fax: (+86) 451 86282195

Table S1. Comparison of performance of PEMF-MMFCs and PEMF-OFF-MMFCs.

MFC	Open circuit voltage (V)	Maximum power density (W/m^2)	Internal resistance (Ω)
PEMF-MMFC -1	0.68	0.94	69.6
PEMF-MMFC -2	0.64	1.02	64.1
PEMF-OFF-MMFC-1	0.68	0.75	87.2
PEMF-OFF-MMFC-2	0.68	0.74	88

Table S2. Similarity-based OTUs and species richness and diversity indices at a threshold of 97%.

Sample	Effective Reads	OTU	Shannon	Simpson	Chao1	ACE	Good's Coverage
PEMF-MMFC -1	59939	146	1.863	0.456	160.53	161.94	0.999
PEMF-MMFC -2	52530	141	1.538	0.385	155.25	149.75	1.000
PEMF-OFF-MMFC -1	50425	149	2.499	0.597	154.06	155.64	1.000
PEMF-OFF-MMFC -2	49027	164	2.589	0.626	187.08	178.80	0.999
PEMF-MMFC -1[a]	44160	114	1.738	0.436	120.00	124.66	1.000
PEMF-MMFC -2[a]	40018	95	1.400	0.365	96.65	98.14	1.000
PEMF-OFF-MMFC -1[a]	39928	115	2.160	0.552	126.38	126.38	1.000
PEMF-OFF-MMFC [a]	37730	128	2.324	0.598	152.92	152.92	0.999

[a] represent sequencing of archaeal 16S rRNA gene amplicons

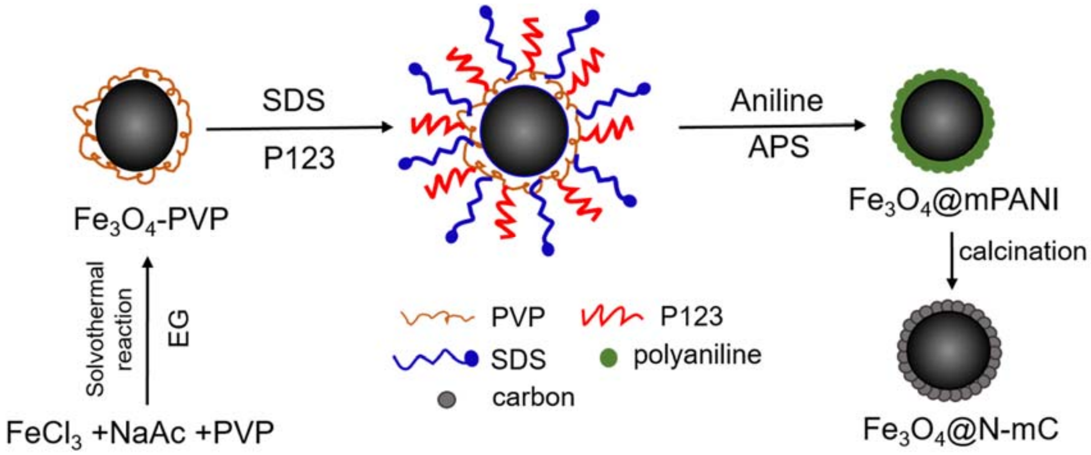

Figure S1. Schematic illustration of the synthesis of Fe_3O_4@N-mC composite.

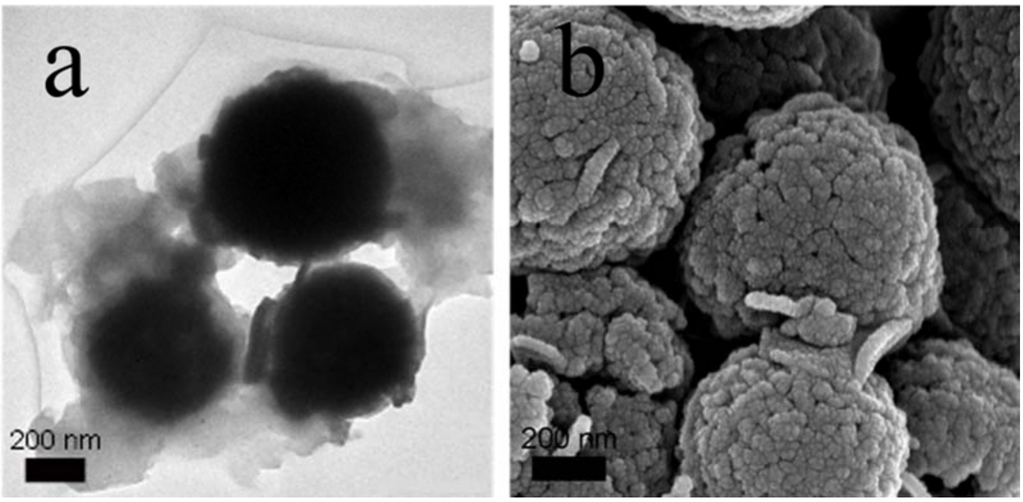

Figure S2. TEM (a) and SEM (b) images of synthesized Fe_3O_4@N-mC composite.

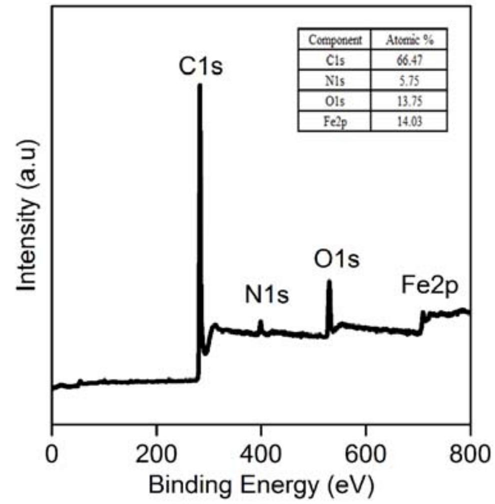

Figure S3. Survey XPS spectra of synthesized Fe3O4@N-mC composite. The insert represents element contents.

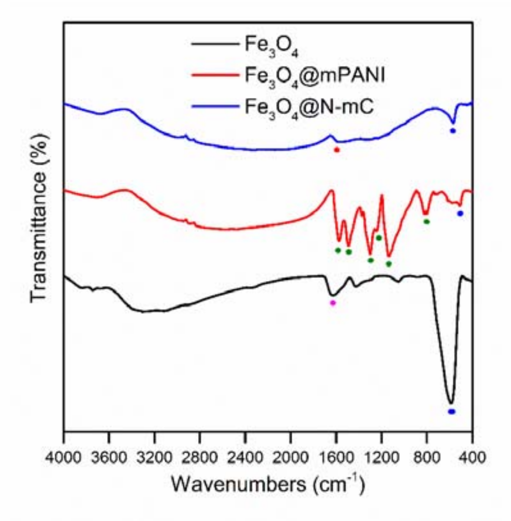

Figure S4. FTIR spectra of Fe3O4, Fe3O4@mPANI and Fe3O4@N-mC composite.

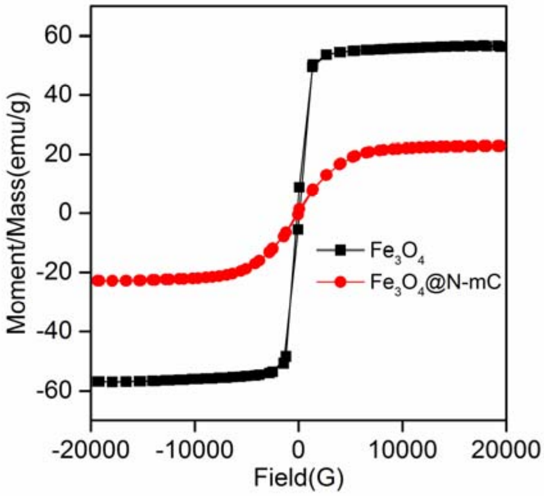

Figure S5. Magnetic hysteresis loops of Fe3O4 and of synthesized Fe3O4@N-mC.
